# Lowering the limit: reducing the CD4 T-cell threshold for ophthalmic screening in patients with HIV in an ethnically diverse UK population

**DOI:** 10.2147/OPTH.S67493

**Published:** 2014-10-03

**Authors:** Rupal Morjaria, Vaneeta Sood, Kaveh Manavi, Alastair K Denniston, Helen Palmer

**Affiliations:** 1 Queen Elizabeth Hospital Birmingham, University Hospitals Birmingham National Health Service Foundation Trust, Birmingham, United Kingdom; 2 Nuffield Department of Ophthalmology, Oxford University Hospitals National Health Service Trust, Oxford, United Kingdom; 3 Centre for Translational Inflammation Research, University of Birmingham, Birmingham, United Kingdom

**Keywords:** cytomegalovirus retinitis, HIV, HAART

## Abstract

**Background:**

Before highly active antiretroviral therapy, cytomegalovirus (CMV) retinitis was a major threat to vision in individuals with HIV. We investigate whether ophthalmic screening of asymptomatic HIV patients still has value in the highly active antiretroviral therapy era and consider CD4 thresholds in line with the world literature and UK experience.

**Methods:**

A retrospective chart review was conducted of all patients seen by the HIV Ophthalmic Service of a UK university hospital both before (2007–2008) and after (2011–2012) introduction of a threshold of CD4 lower than 100 cells/mm^3^. Data collected included CMV and HIV RNA load, CD4 cell counts and CD4 percentage, CMV-immunoglobulin G status, ocular symptoms, and evidence of HIV-related ocular disease.

**Results:**

In total, 54 patients were referred to the HIV ophthalmic service. Three patients failed to attend, resulting in complete data for 51 patients (n=24 for 2007–2008; n=27 for 2011–2012). Seven patients had ophthalmic manifestations of their HIV; these cases had lower CD4 counts than those with normal examinations (median [interquartile range], 9 [7–80] versus 175 [44–394]; *P*=0.0039; Mann–Whitney test). Six cases had HIV retinopathy without sight loss; one case had sight-threatening CMV retinitis associated with a CD4 count of 6 cells/mm^3^.

**Conclusion:**

Before 2008, our practice was to screen all asymptomatic patients with CD4 counts lower than 200 cells/mm^3^. Screening asymptomatic patients with CD4 counts below 100 cells/mm^3^ was not associated with any missed or late-presenting cases of CMV retinitis in our HIV population.

## Introduction

Cytomegalovirus (CMV) retinitis is the most common ocular opportunistic infection in patients with AIDS,^1^,^2^ despite widespread availability of highly active antiretroviral therapy (HAART).^3^ HAART combines the use of three antiretroviral drugs, including a protease inhibitor.^4^ Before the advent of HAART, the 4-year cumulative risk of developing CMV retinitis in patients with HIV was around 25%,^5^,^6^ whereas more recent estimates suggest it is now nearer 7%.^6^–^8^ At present, late diagnosis of HIV is the main reason for CMV retinitis in HIV-infected individuals. The pattern and incidence of HIV-related ophthalmic presentations has changed in the post-HAART era, with few studies reporting them.^9^ Bekele et al found that 39.7% of patients with a CD4+ cell count lower than 200 cells/mm^3^ had ocular manifestations of HIV/AIDS in Jimma, Ethiopia, compared with 20.9% in those with counts higher than 200 cells/mm^3^.^9^

CMV retinitis causes severe retinal damage with a major effect on vision (notably field loss) and with a high risk of recurrence and/or complications such as retinal detachment or immune recovery uveitis. Although a number of clinical phenotypes occur, CMV retinitis typically induces a progressive whitening of the retina around the vessels, with hemorrhages and necrosis described as occurring in a “brushfire” pattern. This is often associated with a mild vitreous inflammation and can progress at the end stage to a rhegmatogenous retinal detachment, causing a dramatic visual decline.

The presence of CMV retinitis is also predictive of disseminated CMV disease and high mortality and is sight-threatening.^1^,^2^ A number of previous studies have indicated that HIV-associated CMV retinitis occurs more commonly in patients with absolute CD4 counts of 50 cells/mm^3^ or less at the time of diagnosis,^5^,^6^ although cases occurring at higher levels are also reported.^4^,^7^,^10^

Baseline CD4 cell count is, however, generally considered a critical parameter in assessing risk for CMV retinitis. The value of prophylactic ophthalmic screening in asymptomatic patients with HIV is uncertain, although where screening is undertaken, the CD4 level usually directs it. Other ocular complications of HIV that may be observed in this population include HIV-associated retinopathy, ocular toxoplasmosis, immune-recovery uveitis, and progressive outer retinal necrosis. In this retrospective study, we present the screening outcomes of patients referred from the HIV clinic for ophthalmic assessment for two time periods: before and after the reduction of our screening threshold for asymptomatic patients with HIV to fewer than 100 CD4 cells/mm^3^. The primary aim of the study was to investigate whether ophthalmic screening of asymptomatic HIV patients has value in the United Kingdom in the HAART era, and if so, whether it can be targeted to particular at-risk groups.

## Methods

### Study population

A retrospective chart review was conducted of all patients seen by the HIV Ophthalmic Service of the University Hospitals Birmingham National Health Service Foundation Trust over the course of two periods: 2007–2008 and 2011–2012. During the first period, the CD4 threshold for referral and review of asymptomatic patients was CD4 lower than 200 cells/mm^3^. The results of the 2007 survey appeared to show no value in screening those with CD4 count greater than 100 cells/mm^3^, and this was therefore revised in line with local and international experience.^5^,^11^ In the second period, a new threshold of lower than 100 cells/mm^3^ was used, and the effect on the effectiveness of screening was assessed. Ophthalmic review included clinical history and full ophthalmic examination, including dilated funduscopy. All blood was processed at a single laboratory (Department of Clinical Immunology, University of Birmingham, United Kingdom). CD4 counts were measured using Becton Dickinson (BD) MultiTEST CD3 FITC/CD8 PE/CD45 PerCP/CD4 APC reagent and BD FACSCanto four-color flow cytometry. TruCOUNT Tubes and simultaneous forward and side scatter were used to obtain percentages and calculate absolute CD4 counts.

Data collected included CMV viral load, CD4 cell count, CD4% (percentage of total lymphocytes that are CD4 T-cells), HIV RNA load, CMV immunoglobulin G, ocular symptoms, and evidence of any HIV-related ocular disease. A retinal consultant with expertise in HIV (HP) confirmed all clinical assessments.

### Data analysis

Frequencies, means and medians of demographics, and clinical and laboratory characteristics were calculated. Comparisons were done for patients referred during the two study periods. Categorical variables were tested with Fisher’s exact test. Continuous nonparametric variables were tested with the Mann–Whitney test, and parametric variables were tested with Student’s *t*-test. A *P*-value less than 0.05 was used to indicate statistical significance. Data analysis was performed using GraphPad Prism (version 5.00).

## Results

In total, over the course of two periods, 54 patients were referred to the HIV Ophthalmic Service: 26 patients during 2007–2008 and 28 patients during 2011–2012. Three patients failed to attend their ophthalmology reviews (two of 26 for 2007–2008 and one of 28 for 2011–2012), resulting in complete data for 51 patients (Table 1). During both periods, the majority of referrals were male (71% versus 74%; not significant; Fisher’s exact test). The proportion of patients who were African or Afro-Caribbean increased during the second period (29% versus 60% [*P*=0.048] for African/Afro-Caribbean versus all other ethnicities; Fisher’s exact test).

Patients in the second study period had overall lower CD4 counts (median, 253 versus 62; *P*=0.034; Mann–Whitney test), with higher HIV RNA copy numbers (median, 19,990 versus 51; *P*=0.0079; Mann–Whitney test). However, there was no significant difference when the groups were compared on the basis of identical screening criteria (ie, with the exclusion of the asymptomatic group of CD4 higher than 100 cells/mm^3^ but lower than 200 cells/mm^3^ seen during the first, but not the second, study period). Rates of CMV immunoglobulin G positivity were similar in the two groups (75% and 78%; not significant; Fisher’s exact test).

In terms of ocular disease, a total of seven of 51 patients had ophthalmic manifestations of their HIV (Table 2). This included three of 24 patients in 2007 and four of 27 patients in 2011. All cases were asymptomatic at time of ophthalmic screening. In most cases (six of seven), the ocular disease was HIV retinopathy and was visually nonsignificant. In one of seven cases, bilateral CMV retinitis was noted, which was treated with an induction course of intravenous ganciclovir followed by maintenance oral valganciclovir. Unfortunately, this patient did not adhere to any treatment after 6 months, and the patient suffered a severe recurrence of CMV retinitis and died 11 months later. This patient was the only one of the seven patients with ocular disease to have died during the follow-up period (up to 7 years for group 1 and up to 3 years for group 2).

The CD4 count in those patients with HIV-associated ophthalmic disease was significantly lower than in those with normal examinations, with a median (interquartile range) of nine (7–80) versus 175 (44–394) (*P*=0.0039; Mann–Whitney test). The median HIV count in those patients with HIV-associated ophthalmic disease was significantly higher than in those with normal examinations, with a median (interquartile range) of 987,151 (568,025–997,270) versus 470 (40–36,852) (*P*=0.0019; Mann–Whitney test). The one case with CMV retinitis (representing 2% of all patients screened) had a CD4 count of 6 cells/mm^3^ (CD4% =3) at the time of screening. This was the lowest recorded CD4 count in our series; the peripheral blood CMV DNA copy number in this patient was 1.5×10^4^ copies/mm^3^. There were no “late referrals” of patients with HIV-associated ophthalmic disease, and in particular no other cases of HIV-associated CMV retinitis, presented to the ophthalmic service during these periods. No cases of immune recovery uveitis were seen in our cohort.

## Discussion

As the CD4 T-lymphocyte is the primary target of HIV, the CD4 count is commonly used as a surrogate laboratory indicator of the degree of immunocompromise and potential risk of opportunistic infections, including CMV retinitis. CMV retinitis is frequently diagnosed late because of the lack of visual symptoms. Before 2008, our practice was to screen all asymptomatic patients with CD4 counts lower than 200 cells/mm^3^. Our review in 2007–2008 of the cohort of 24 patients seen found only one patient with CMV retinitis; this patient had a CD4 count of 6 cells/mm^3^. In light of this, and supported by other studies worldwide, we modified our screening criteria to include asymptomatic patients with CD4 counts lower than 100 mm/μL. This report shows that during a subsequent study period, the introduction of the new screening criteria was not associated with any missed or late-presenting cases of CMV retinitis in our HIV population. Indeed, since the introduction of the more stringent screening criteria (ie, from January 1, 2011, to the time of writing this paper), we have had no cases of HIV-associated CMV retinitis that would not have been picked up by these criteria.

Our adoption of a lower cutoff for asymptomatic patients is also supported by the large Longitudinal Study of Ocular Complications of AIDS (LSOCA).^6^ In this multicenter prospective observational study, patients older than 13 years with a diagnosis of HIV were recruited from specialist ophthalmic clinics in the United States. The study was initiated in 1998, and as of December 31, 2009, it had enrolled 2,271 participants. Of these, 492 patients had CMV retinitis in at least one eye at enrollment. By looking at 1,600 patients who did not have CMV retinitis at baseline and who had 6 months or longer of follow-up, the investigators found 29 incident cases during 8,134 person-years of follow-up. This translates into an incidence rate of CMV retinitis in individuals with AIDS of 0.36/100 person-years. The investigators note that a CD4 count lower than 50 cells/mm^3^ was the single most important risk factor for the development of CMV retinitis, with a hazard ratio of 136 (95% confidence interval, 30–605) and an incidence rate of 3.89/100 person-years.

The increased risk associated with a CD4 count lower than 50 cells/mm^3^ has been observed in a number of studies, including some from the pre-HAART era. In 1992, Pertel et al^6^ reported that based on Kaplan–Meier survival curves, the percentage of patients developing CMV retinitis by 27 months with baseline CD4 lymphocyte counts of 0–50, 51–100, and 101–250 cells/mm^3^ was 41.9%, 26.3%, and 14.7%, respectively (log-rank test, *P*=0.003). In support of this, Lai et al report that patients with baseline CD4 cell count lower than 100 cells/mm^3^ were significantly associated with the presence of CMV retinitis (*χ*^2^-test, *P*=0.013).^4^ Gharai et al reviewed 199 eyes in HIV-infected patients and found that the median CD4 count of the patients with active CMV retinitis was 75 cells/mm^3^.^12^

In our study, we noted that only one of seven patients with HIV-associated ophthalmic disease was receiving HAART before referral for ophthalmic screening. This was because most of the patients were newly diagnosed or had a known diagnosis but were new to the United Kingdom; those cases who were not taking HAART before ophthalmic review were started on HAART by the HIV service immediately after ophthalmic review. Previous studies have observed improved prognosis in CMV retinitis patients receiving HAART, specifically in terms of reduced progression, detachment, and visual loss.^11^,^13^–^15^ In the LSOCA report by Sugar et al,^7^ the investigators comment that 26 of the 29 patients with CMV retinitis and a CD4 count lower than 50/cells/mm^3^ had been noted to have been receiving HAART at the clinic review immediately before the ophthalmic assessment, suggesting there was either treatment failure or lack of adherence.^6^

It should be noted, however, that CMV retinitis may occur in patients with CD4 counts higher than 50 cells/mm^3^, and that our screening criteria would therefore miss these individuals. Jacobson et al^10^ reported five cases in whom the CD4 counts were higher than 195 cells/mm^3^ at the time of diagnosis of CMV retinitis. Of interest, however, is that all five cases had CD4 counts lower than 85 cells/mm^3^ at baseline (which was within the previous 6 months), and three of the five had CD4 counts lower than 50 cells/mm^3^. The LSOCA study noted one incident case of CMV retinitis with a CD4 count in the 50–99 cells/mm^3^ range (incident rate, 0.18/100 person-years) and two cases in the higher than 100 cells/mm^3^ group (incident rate, 0.03/100 person-years).^6^ Again, it should be noted that these counts refer to the CD4 level at the visit immediately preceding their ophthalmic assessment and that all three cases had recorded levels lower than 50 cells/mm^3^ at some earlier point in their disease.

CMV retinitis is a blinding condition, and our patient with CMV retinitis lost her vision to hand movements despite treatment with high dose intravenous and intravitreal ganciclovir. Intravitreal ganciclovir is a useful treatment for CMV retinitis, as it avoids the adverse effects and high doses from low bioavailability of systemic treatment.^16^ However, treatment requires monthly appointments and close follow-up in a group of patients at high risk for nonattendance. Appropriate screening and early detection are therefore of upmost importance.

There was a significant increase in the African/Afro-Caribbean population between the first and second screening periods, reflecting overall changes in the Birmingham population. Census data show that although during the last decade the proportion of people in Birmingham identifying themselves as Afro-Caribbean has fallen slightly, going from 4.9% to 4.5% (2001 versus 2011 Census data), the proportion of people identifying themselves as African has increased from 0.6% to 2.8%.^17^ This could be as a result of a number of factors. A recent study across 1,111 countries evaluated the role of poverty in racial/ethnic disparities in HIV prevalence.^18^ It found that racial/ethnic disparities remained in nonurban areas, but there was not a significant difference in urban areas when controlled for poverty.^18^

We recognize that even though our study was conducted within a large urban population (all of South Birmingham, United Kingdom), the actual cohort is relatively small and cannot be used to provide incident data in line with the LSOCA study. This is, of course, to be welcomed, as it reflects the dramatic health improvement in the HIV population since the advent of HAART. As CMV retinitis becomes less common, accurate estimates for its incidence and prevalence in the United Kingdom will depend on national surveys (such as conducted through the British Ophthalmic Surveillance Unit), networks, or the establishment of a national registry for such cases. This last option would also provide the opportunity for prospective standardized capture of data similar to that adopted by the LSOCA, in contrast to the retrospective nature of ours and most other studies.

CMV retinitis is a potentially avoidable, blinding condition. Patients with low CD4 counts are often asymptomatic. Our data support the findings of other studies: that in patients taking HAART, CMV retinitis is increasingly rare, and when it does occur, it is usually in patients with a CD4 cell count lower than 50 cells/mm^3^. Given the low frequency of CMV retinitis and the cost of screening, it may be argued that ophthalmic screening of asymptomatic HIV patients is not necessary. We would argue that the devastating effect of CMV retinitis, its rapid asymptomatic progression, and the availability of effective treatment sufficiently justify screening in the highest-risk groups. On the basis of studies such as the LSOCA,^6^ and supported by our local experience, we argue that a CD4 count lower than 100 cells/mm^3^ is a reasonable threshold to use for screening asymptomatic patients with known HIV.

## Figures and Tables

**Table 1:** Comparison between patients screened during the two study periods

Characteristic	Screening period 1 (2007–2008)	Screening period 2 (2011–2012)	*P*-value
Number of patients	24	27	
Sex, n (%)			1.00*
Male	17 (71)	20 (74)	
Female	7 (29)	6 (26)	
Ethnicity, n (%)			0.048*
Caucasian	13 (54)	9 (33)	
African/Afro-Caribbean	7 (29)	16 (60)	
European	4 (17)	2 (7)	
Asian	0 (0)	0 (0)	
CD4 count (cell/mm^3^), n (%)			0.034**
0–50	5 (19)	11 (40)	
51–100	3 (11.5)	7 (26)	
101–200	3 (11.5)	2 (7)	
>200	13 (50)	7 (26)	
No result	2 (8)	0	
Median	253	62	
CD4%			0.274**
Median (interquartile range)	18.0 (8.8–26.1)	10 (4–22.0)	
HIV RNA, copies/mm^3^			0.0079**
Median (interquartile range)	51 (40–7,891)	19,990 (188–627,525)	
Cytomegalovirus immunoglobulin G (%)			1.00*
Positive	18 (75)	21 (78)	
Negative	6 (25)	6 (22)	
Ophthalmic manifestations of HIV, n (%)			1.00*
Present	3 (13)	4 (15)	
Not present	21 (87)	23 (85)	

**Notes:**

* Fisher’s exact test of (1) male versus female, (2) African/Afro-Caribbean versus all other ethnicities, (3) cytomegalovirus immunoglobulin G-positive versus immunoglobulin G-negative, and (4) ophthalmic manifestations of HIV present versus not present.

** Mann–Whitney test.

**Table 2:** Characteristics of patients diagnosed with cytomegalovirus retinitis and HIV retinopathy

Characteristic	Patient 1	Patient 2	Patient 3	Patient 4	Patient 5	Patient 6	Patient 7
CD4 count (cells/mm^3^)	7	77	6	82	80	9	8
HIV viral load (copies/mm^3^)	38,147	429,196	Not known	Not known	999,764	984,513	989,788
Highly active antiretroviral therapy at time of ophthalmic assessment	Yes	No	No	No	No	No	No
Symptoms at presentation	None	None	None	None	None	None	None
Clinical findings	Cotton wool spots	Cotton wool spots	Retinal necrosis, hemorrhages	Cotton wool spots, telangiectasia	Cotton wool spots	Cotton wool spots, blot hemorrhages	Cotton wool spots
Unilateral/bilateral disease	Bilateral	Bilateral	Bilateral	Bilateral	Bilateral	Bilateral	Bilateral
Ocular diagnosis	HIV retinopathy	HIV retinopathy	Cytomegalovirus retinitis	HIV retinopathy	HIV retinopathy	HIV retinopathy	HIV retinopathy
Ophthalmic treatment	Observation only	Observation only	Intravenous ganciclovir induction then maintenance oral valganciclovir plus intravitreal ganciclovir (low compliance to treatment)	Observation only	Observation only	Observation only	Observation only
Outcome	No visual consequences	No visual consequences	Visual loss to hand movements (RE); six of nine LE; patient died 17 months after ophthalmic presentation	No visual consequences	No visual consequences	No visual consequences	No visual consequences

**Abbreviations:** RE, right eye; LE, left eye.
